# The use of *maoto* (*Ma-Huang-Tang*), a traditional Japanese Kampo medicine, to alleviate flu symptoms: a systematic review and meta-analysis

**DOI:** 10.1186/s12906-019-2474-z

**Published:** 2019-03-18

**Authors:** Tetsuhiro Yoshino, Ryutaro Arita, Yuko Horiba, Kenji Watanabe

**Affiliations:** 1 Center for Kampo Medicine, Keio University School of Medicine, 35 Shinanomachi, Shinjuku-ku, Tokyo, 160-8582 Japan; 2 Linus Pauling Institute, Oregon State University, Linus Pauling Science Center, Corvallis, OR USA; 3 Department of Education and Support for Regional Medicine, Department of Kampo Medicine, Tohoku University School of Medicine, 1-1 Seiryo, Aoba-ku, Sendai, Miyagi Japan; 4 Faculty of Environment and Information Studies, Keio University, 5322 Endo, Fujisawa, Kanagawa Japan

**Keywords:** Influenza, Japanese traditional herbal medicine, Symptomatic treatment, Neuraminidase inhibitors

## Abstract

**Background:**

Influenza is a common viral infection worldwide. *Maoto* (*ma-huang-tang*) was developed in ancient China and is used to alleviate flu symptoms. Currently, no meta-analyses have evaluated the efficacy and safety of *maoto* for alleviating flu symptoms.

**Methods:**

In the present study, we searched MEDLINE/PubMed, the Cochrane Central Register of Controlled Trials (CENTRAL), EMBASE, a Japanese database (*Ichushi*), two Chinese databases (China National Knowledge Infrastructure and VIP), and two Korean databases (Korean Medical database and Korean Association of Medical Journal Editors) for studies published in or before October 2017. Clinical studies that compared *maoto* plus neuraminidase inhibitors (NAIs) vs. NAIs alone, or *maoto* alone vs. NAIs alone, were included in the present analysis. The primary outcome measure (efficacy) was the length of time from the start of medication to resolution of influenza symptoms (fever, headache, malaise, myalgia, and chills) and virus isolation. The secondary outcome measures (safety) were as follows: (1) side effects and adverse reactions, such as nausea, abnormal behaviour, or discontinuation of symptomatic treatment; (2) morbidity (complications caused by influenza infection) or mortality; and (3) hospitalisation for any reason.

**Results:**

Twelve relevant studies were identified, including two randomised controlled trials (RCTs, *N* = 60) and ten non-randomised studies (NRSs, *N* = 1110). We found that *maoto* plus NAIs was superior to NAIs alone in terms of the duration of fever in one RCT (*P* < 0.05, median difference = − 6 h) and four NRSs (*P* = 0.003, weighted mean difference = − 5.34 h). The duration of symptoms or virus isolation did not differ between *maoto* and NAIs. No severe side effects or adverse reactions were reported related to *maoto* or NAIs.

**Conclusions:**

Although we could not reach a definitive conclusion because of the small sample sizes and high risk of bias in the analysed studies, *maoto* may lower the duration of fever when it is used alone or in combination with NAIs and may be a well-tolerated treatment. More RCTs are needed to determine the efficacy and safety of *maoto*.

**Electronic supplementary material:**

The online version of this article (10.1186/s12906-019-2474-z) contains supplementary material, which is available to authorized users.

## Background

Influenza is a common viral infection worldwide, occurring in seasonal epidemics. According to the World Health Organization (WHO), 5–15% of the population is affected by annual epidemics of influenza infection in the upper respiratory tract [1], and 290,000–650,000 people die from influenza-related diseases annually [2].

Three types of antiviral drugs have been approved by the Japanese national health insurance system for the symptomatic treatment of influenza infection, namely neuraminidase inhibitors (NAIs), adamantanes, and cap-dependent endonuclease inhibitors. Four NAIs—oral oseltamivir, inhaled zanamivir, inhaled laninamivir, and intravenous peramivir—are available and widely prescribed in Japan because they act against both types of the influenza virus, A and B. In contrast, amantadine and rimantadine (known as adamantanes) are less widely prescribed because they only act against the influenza A virus, which shows marked resistance to adamantanes [3]. Baloxavir marboxil is a newly approved cap-dependent endonuclease inhibitor, and as such, little is known about its post-marketing safety. The resistance of influenza to baloxavir marboxil has been reported in 2.2 and 9.7% of the phase 2 and 3 clinical trials, respectively [4].

A recent systematic review of oseltamivir revealed that it can shorten the duration of symptoms by 16.8 h in adults but does not reduce hospital admissions due to complications from influenza. The review also pointed out that the drug conferred an increased risk of nausea and vomiting in adults [5]. The WHO has recommended that patients in low-risk groups should be managed with symptomatic treatments and advised to stay home to avoid spreading the infection. However, patients at high risk of developing severe or complicated illness should be administered antiviral agents [1]. Currently, few influenza strains are resistant to NAIs [6], but antiviral resistance may increase if the widespread use of NAIs is continued, as was observed for oseltamivir-resistant seasonal H1N1 influenza A viruses between 2007 and 2009 [3].

Recently, the efficacies of several traditional Chinese medicine formulas as symptomatic treatments for influenza have been evaluated. For instance, a systematic review of the Cochrane library reported that *Ganmao* capsules were more effective than amantadine at shortening the duration of influenza symptoms [7]. A randomised controlled trial (RCT) showed that *maxingshigan-yinqiaosan*, alone or in combination with oseltamivir, reduced the duration of fever in patients infected with the 2009 H1N1 influenza virus [8].

*Maoto* (*Ma-Huang-Tang* in Chinese) has been widely prescribed as a symptomatic treatment for the common cold and flu according to claims in the Japanese national health insurance system. It can be prescribed to both children and adults. Traditionally, the symptoms that indicate *maoto* are headache, chill, fever, arthralgia, and cough, without sweating. *Maoto* can also be applied for rheumatoid arthritis, bronchial asthma, infant nasal obstruction, and difficulties in sucking milk.

*Maoto* is a multicomponent formulation, originally extracted from four crude drugs, as follows: 5 g of ephedra herb, 5 g of apricot kernel, 4 g of cinnamon bark, and 1.5 g of glycyrrhiza root. It is currently prepared for prescription use in Japan as granules (7.5 g daily, produced by Tsumura & Co., Teikoku Pharma, and Honzo Co.; no official paediatric dosage available) or powder (6.0 g daily, produced by Kracie Pharma and Kotaro Pharm. Co.; no official paediatric dosage available) through the process of decoction, concentration, drying, and the addition of an excipient. The *maoto* preparation is orally administered, usually after dissolution in warm water.

Some studies have demonstrated that *maoto* and its component ingredients are active. For instance, ephedra herb and its tannins inhibit endosome acidification and influenza A virus fusion to the cell membrane [9, 10]. Glycyrrhizin, an active component of glycyrrhiza, reduces the number of cells infected with influenza A and inhibits virus uptake through the cell membrane during the early phase of infection [11]. Cinnamaldehyde, which is derived from cinnamon bark, inhibits protein synthesis by the influenza A virus at the post-transcriptional level. In one study carried out in mice, inhalation and nasal inoculation of cinnamaldehyde increased the survival rate after virus infection [12]. Masui et al. [10] reported that *maoto* acts against influenza A in vitro, while laninamivir and amantadine do not. In addition, using multiple subtypes of the influenza virus (A/PR8, A/H3N2, A/H1N1pdm, and B), the authors found that *maoto* reduced the intracellular virus titre, as well as the levels of matrix protein 2 and nucleoprotein present in the experimental system. Nagai et al. [13] showed that *maoto* (0.8 g/kg/day and 1.3 g/kg/day) had an antipyretic effect in the early phase of influenza A infection in mice and that the levels of anti-influenza immunoglobulin M, immunoglobulin A, and immunoglobulin G1 antibodies increased in nasal fluid, bronchoalveolar lavage, and serum. Thus, *maoto*, as a multicomponent formulation, has multiple effects on the life cycle of the influenza virus and broad influence on host metabolism [14].

*Maoto* is a prescription drug that has been covered by the Japanese national health insurance system for over 40 years. The cost of *maoto* is much less than that of NAIs. Specifically, the officially set drug cost of *maoto* is 150 JPY (1.4 USD) per person, whereas the standard prescription of oseltamivir and acetaminophen costs 3260 JPY (29.6 USD). We previously calculated that, if half a prescription of oseltamivir was replaced with *maoto*, the annual saving in medical costs in Japan would be 9 billion yen (82 million USD) [15]. Nowadays, *maoto* is also available in the pharmacy as an over-the-counter drug, although its concentration is half that of the prescribed medication.

The efficacy and safety of *maoto* in alleviating flu symptoms have been evaluated in clinical studies. These studies compared *maoto* with NAIs, or *maoto* plus NAIs with NAIs alone. However, the results have been inconsistent, and no meta-analysis has yet analysed the efficacy and safety of *maoto* in alleviating flu symptoms.

Thus, the objective of this review and meta-analysis was to evaluate the efficacy and safety of *maoto* in alleviating flu symptoms.

## Methods

### Criteria for inclusion in the present review

We included both RCTs and comparative non-randomised studies (NRSs) of prescription *maoto* extract. The NRSs included quasi-RCTs, non-RCTs, prospective or retrospective cohort studies, historically controlled trials, and (nested) case-control studies [16].

We included studies that enrolled patients who had uncomplicated influenza diagnosed using a rapid antigen detection test (RADT) and/or genetic detection with polymerase chain reaction. Studies were included regardless of the patients’ age.

We included studies assessing the efficacy of *maoto*. The possible comparisons were as follows: (1) *maoto* vs. placebo, (2) combination of *maoto* plus NAIs vs. NAIs alone, and (3) *maoto* vs. NAIs. Co-interventions were allowed if they were offered to both arms of the study. We excluded studies that included other herbal formulas.

The primary outcome measure (efficacy) was the length of time from the start of medication to resolution of influenza symptoms (fever, headache, malaise, myalgia, and chills) and virus isolation.

The secondary outcome measures (safety) were as follows: (1) side effects and adverse reactions, such as nausea, abnormal behaviour, or discontinuation of symptomatic treatment; (2) morbidity (complications caused by influenza infection) or mortality; and (3) hospitalisation for any reason.

### Search methods for identification of studies

We searched the following databases for studies published in or before October 2017: MEDLINE/PubMed, the Cochrane Central Register of Controlled Trials (CENTRAL), EMBASE, a Japanese database (*Ichushi*), two Chinese databases (China National Knowledge Infrastructure [CNKI] and VIP), and two Korean databases (Korean Medical database [KMbase] and Korean Association of Medical Journal Editors [KAMJE]). In this search, we used the key words ‘maoto/mao-to/ma-huang-tang’, and ‘flu/influenza’. Our search strategies are supplied in Additional file 1: Search Strategies and Results. We did not use any publication or language restrictions. All reference lists were checked to identify additional studies.

### Data collection and analysis

The first two authors (TY and RA) scanned the titles, abstracts, and keywords of every record retrieved. We located the full articles for further assessment when the information given suggested that the study (1) included patients with uncomplicated influenza and (2) assessed the efficacy of *maoto* using one or more relevant clinical outcome measures.

Two authors (TY and RA) independently extracted, checked, and made a review table of the articles. The extracted data comprised the (1) authors and title of the study, (2) year of publication, (3) study size, (4) age and sex of participants, (5) detailed methodological information, (6) dose and duration of the intervention, (7) details of control interventions, (8) outcomes, and (9) side effects or adverse reactions. TY entered these data into the Review Manager software.

We assessed the reporting quality of each study, basing the assessment largely on the quality criteria specified by the Cochrane Handbook for Systematic Reviews of Interventions [16] for RCTs and the Risk of Bias in Non-randomized Studies of Interventions [17] for NRSs. The authors resolved any differences of opinion by discussion. Funnel plots were inspected visually to assess the possibility of publication bias.

We performed a quantitative meta-analysis when data for a given outcome were available in more than two of the included studies, regardless of the daily dosage or duration of *maoto*. To evaluate symptom duration, we ignored differences in the definition of symptom, as well as differences in the viral type (A or B) or surface antigen (e.g. H1N1 or H3N2). The unit of analysis was the individual participants. We tried to obtain any relevant missing data from the study authors by e-mail or telephone.

The present meta-analysis was performed using Review Manager (RevMan) Ver. 5.3 for Mac (Cochrane Collaboration: https://community.cochrane.org/help/tools-and-software/revman-5).

We examined heterogeneity through visual inspections of forest plots, a standard χ^2^ test with a significance level of α = 0.1, and the I^2^ statistic (an I^2^ statistic ≥75% indicated considerable heterogeneity). This analysis quantified inconsistency across studies and allowed us to assess the effects of study heterogeneity on the meta-analysis. To combine studies, we used the fixed-effects model when the studies in a given subgroup were significantly similar (*P* > 0.10; I^2^ statistic < 75%). We included RCTs and NRSs in this meta-analysis, and RCTs and NRSs were always analysed separately. The overall effect was tested using the Z score, with *P* < 0.05 being considered statistically significant.

When heterogeneity was suggested by the χ^2^ test and I^2^ statistic, we used a random-effects model to analyse whether heterogeneity may have led to the different effects among studies. In this model, data should be continuous, thus we used the weighted mean difference and 95% confidence intervals to analyse data that had been measured using the same scale, and then combined the weighted mean differences (e.g. duration of fever). We attempted to determine the potential causes of heterogeneity by examining the individual study and subgroup characteristics.

## Results

### Description of studies

In the present review, 241 studies were found in the Japanese database (*Ichushi*), 11 in MEDLINE, 11 in EMBASE, 11 in CENTRAL, 6 in VIP, 7 in CNKI, and one by a Google hand search. We did not find any relevant studies in KMbase or KAMJE. After removing 24 duplicates, another 239 records were removed because they were (1) not relevant comparative clinical studies of *maoto*, (2) basic research, (3) reviews, or (4) case reports. Subsequently, we excluded 12 full-text articles of clinical studies that (1) lacked relevant outcomes (*N* = 5), (2) involved different interventions (*N* = 3, *maoto* + other herbal formulas), or (3) duplicated participants (*N* = 4, same data presented in another included study).

**Fig. 1:**
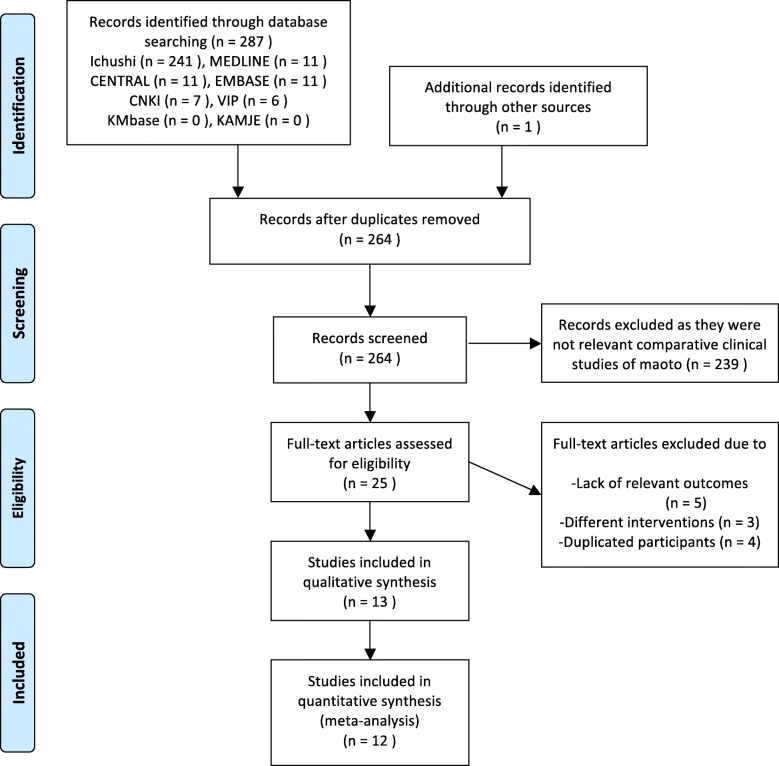
PRISMA 2009 flow diagram

Ultimately, 13 studies fulfilled our inclusion criteria [18–30]. However, only 12 of the 13 studies were included in our meta-analysis (Fig. 1) because one study reported the mean difference in patients’ body temperature on different days after medication administration rather than the total mean or median fever duration [26], and although we contacted the authors, we could not obtain the raw data. Among the 12 studies, three were published by Kawamura [19, 21, 23] and two were published by Nabeshima et al. [25, 29]. The full text of these articles was checked carefully to avoid duplication of participants. Details of the included studies are shown in Additional file 2: Table S1.

None of the studies compared the effects of *maoto* with those of a placebo. Among the 12 studies, we found two NRSs comparing the effects of *maoto* with no treatment [18, 30], but no further analyses were performed. Among the 12 studies, we found one RCT [17] and four NRSs [18, 22, 28, 30] comparing the effects of *maoto* plus NAIs with those of NAIs alone. We also identified one RCT [29] and eight NRSs [19, 21, 23–25, 27, 28, 30] comparing the effects of *maoto* alone with those of NAIs. Two of the studies assessed both the effects of *maoto* plus NAIs and those of *maoto* alone [28, 30]. One RCT [20] also assessed both the effects of *maoto* plus NAIs and those of *maoto* alone. However, some of the participants allocated to the *maoto* only group were not virologically diagnosed with an influenza infection, and thus we excluded the *maoto* only group in that RCT from this review.

All of the participants in the included studies had been diagnosed with influenza using RADT. In some studies, genetic detection with polymerase chain reaction or virus culture was added to confirm the diagnosis.

In most of the included studies, *maoto* extract granules produced by Tsumura Co. (Tokyo, Japan) were used as an intervention. The reason might be that the influenza infection is clearly indicated only for *maoto* produced by Tsumura Co. in Japan. No self decoctions from crude drugs were used in any of the studies.

**Fig. 2:**
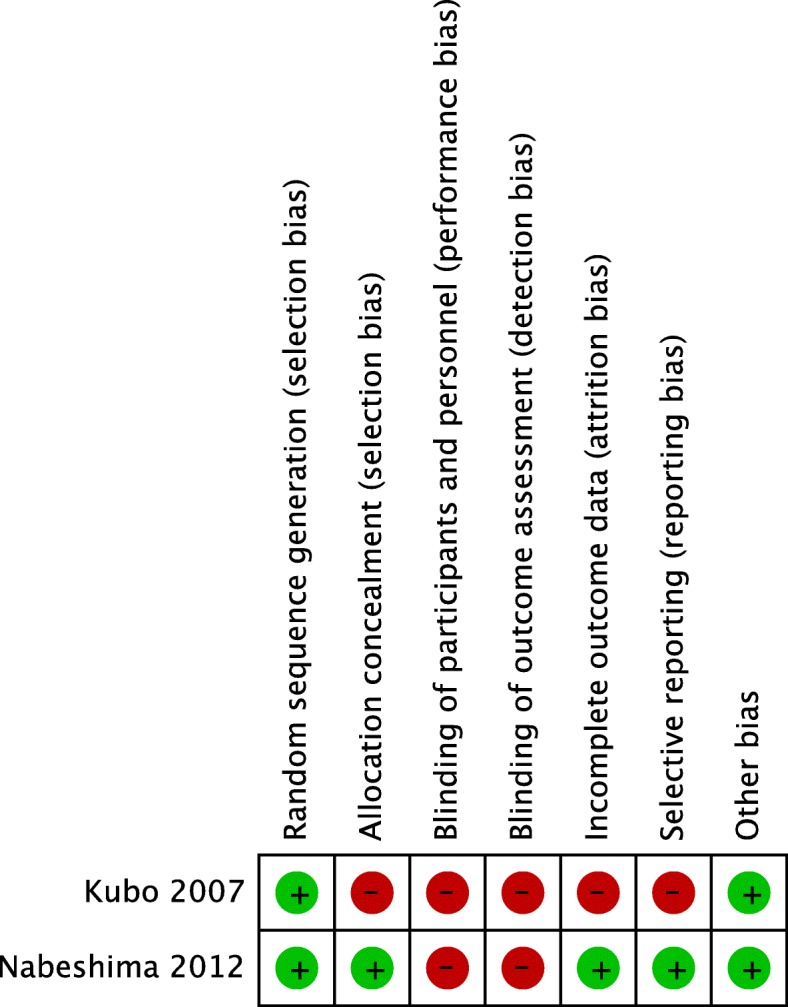
Risk of bias summary for randomised clinical studies. Green and red circles indicate a low and high risk of bias, respectively

Among the 12 studies, there were two RCTs [20, 29] and ten NRSs, including one quasi-RCT [27] and nine cohort studies. The overall risk of bias is presented and summarised graphically in Fig. 2 for the RCTs and in Table 1 for the NRSs. No double-blinded RCTs were identified by our literature search. One study had a retrospective cohort study design; it reported demographic background information for all of the participants but failed to divide them in terms of the drug type used [28]. Five of the studies lacked any mention of excluded patients [19, 21–23, 27]. All of the studies evaluated the duration of fever, but few studies evaluated other symptoms. Four of the studies failed to report adverse events [21, 23, 24, 27]. Only one study with a pre-defined protocol was available [29]. Funnel plots are shown in Additional files 3, 4, and 5: Figures S1–S3. One study reported the duration of fever in days rather than hours [18], and hence only a rough duration could be recorded.

**Table 1:** Risk of bias summary for non-randomised studies

Study	Confounding	Participant selection	Classification of intervention	Deviations from intended Interventions	Incomplete outcome data	Measurement of outcomes	Selective reporting	Overall
Fukutomi 2005 [18]	3	1	3	1	0	1	1	3
Taketani 2008 [22]	3	1	3	1	0	1	1	3
Tsuji 2011 [28]	3	1	1	1	0	1	1	3
Toriumi 2012 [30]	2	1	3	1	3	1	1	3
Kawamura 2007 [19]	3	1	3	1	2	1	1	3
Kawamura 2008 [21]	3	1	3	1	0	1	1	3
Kawamura 2009 [23]	3	1	3	1	0	1	1	3
Mori 2010 [24]	3	1	3	3	0	1	3	3
Nabeshima 2010 [25]	2	1	1	1	3	1	1	3
Suzuki 2011 [27]	2	1	1	1	2	1	1	2

0: No information, 1: Low, 2: Moderate, 3: Serious, 4: Critical

### Effects of interventions

#### The combination of maoto plus NAIs vs. NAIs alone

The duration of overall symptoms did not differ between patients administered *maoto* plus NAIs and those administered NAIs alone according to an NRS by Tsuji et al. [28].

**Fig. 3:**
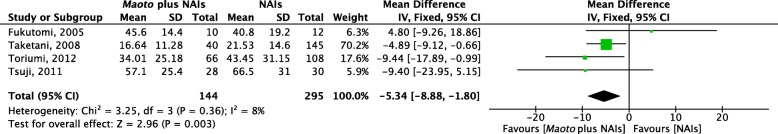
Forest plot of fever duration after drug administration: *maoto* plus neuraminidase inhibitors (NAIs) vs. NAIs alone. The combination of *maoto* plus NAIs is superior to NAIs alone (*P* = 0.002, weighted mean difference = −5.41 h, I^2^ = 0%). The heterogeneity is not significant (P > 0.1, I^2^ < 75%), and a fixed-effects model is used for this outcome

The combination of *maoto* plus NAIs was superior to NAIs alone in terms of the duration of fever in one RCT (*P* < 0.05, median difference = − 6 h; according to their analysis) and four NRSs (*P* = 0.003, weighted mean difference = − 5.34 h) (Fig. 3). For the meta-analysis of the four NRSs, the heterogeneity was not significant (*P* = 0.36, I^2^ = 8%), and we chose a fixed-effects model for this outcome.

One NRS by Fukutomi et al. [18] reported the duration of headache and malaise, and found a significantly shorter duration of headache (− 1.1 day, *P* = 0.018; according to their analysis) in the *maoto* plus NAIs group.

We did not identify any articles mentioning the dosing times of acetaminophen or the duration of virus isolation after symptomatic treatment.

### Maoto vs. NAIs

**Fig. 4:**
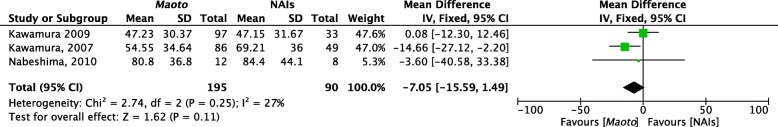
Forest plot of overall symptom duration after drug administration: *maoto* vs. neuraminidase inhibitors (NAIs). The duration of symptoms does not differ significantly between *maoto* and NAIs. The heterogeneity is not significant (*P* > 0.1, I^2^ < 75%), and a fixed-effect model is used for this outcome. Kawamura [19, 23] defined the duration of symptoms as the time until complete disappearance of the symptoms, while Nabeshima et al. [25] defined it as the time during which the symptom score was over the cut-off point for symptoms

The duration of overall symptoms did not differ between patients administered *maoto* and those administered NAIs in either one RCT or three NRSs (Fig. 4). The heterogeneity of the included NRSs was not significant (*P* = 0.25, I^2^ = 27%), and we chose a fixed-effects model for this outcome. Our search for studies comparing *maoto* vs. NAIs identified four reports from two first authors, Kawamura [19, 23] and Nabeshima [25, 29]. However, the definition of the duration of overall symptoms differed between the two authors. Kawamura defined the duration of symptoms as the time until complete disappearance of the symptoms, while Nabeshima et al. defined the duration of symptoms as the time during which the symptom score (rated on a scale of 0 [none] to 3 [severe] daily; the crude scores were added and the sum was used as the symptom score) was over the defined cut-off point (≥2 for their NRS [25] and ≥ 8 for their RCT [29]) for symptoms.

**Fig. 5:**
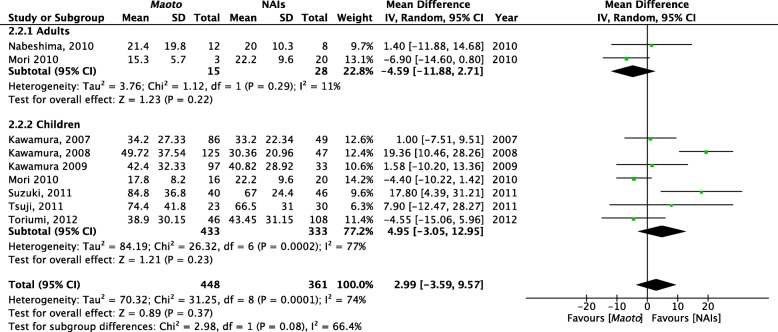
Forest plot of fever duration after drug administration: *maoto* vs. neuraminidase inhibitors (NAIs). The duration of fever does not differ significantly between *maoto* and NAIs. The heterogeneity is significant (*P* < 0.1, I^2^ > 75%), and a random-effects model is used for this outcome. The plot shows three studies by Kawamura [19, 21, 23] and one by Nabeshima et al. [25]. The full-text articles of these studies were carefully checked for duplication of participants

The duration of fever did not differ between *maoto* and NAIs in either the RCTs or NRSs. In an RCT by Nabeshima et al. [29], the authors reported a significantly shorter duration of fever in the *maoto* group than in the oseltamivir group. However, this trial also included participants who were administered zanamivir, and the difference in fever duration was no longer significant when the participants who were administered zanamivir were combined with participants who were administered oseltamivir as the NAIs group. Eight NRSs were included in the meta-analysis of fever duration. The heterogeneity was significant (*P* = 0.0001, I^2^ = 74%), and we chose a random-effects model for this outcome (Fig. 5). As a subgroup analysis, we divided the studies according to the age of the participants (adults and children). This subgroup analysis did not significantly alter the combined results. In the adult subgroup, *maoto* tended to be superior to NAIs (weighted mean difference = − 4.59 h, *P* = 0.22). The tendency was rather opposite in children (+ 4.95 h, *P* = 0.23), and this discrepancy might be caused by two NRSs [21, 27] that included particularly young participants (age < 10 years) (see Additional file 2: Table S1). Further, the authors of these two NRSs did not report data regarding medication compliance, and as it might be difficult for younger participants to adhere to the medication compliance requirements, especially given the strong smell and taste of *maoto*, this may have also contributed to the discrepant results in children. Regarding the daily dosage, although we noticed that the daily dosage of *maoto* used by children was generally less than that used in adults, we did not observe a relationship between the dosage and treatment efficacy in paediatric participants. We did not perform further subgroup analyses, such as age ≤ 10 years vs. > 10 years, viral type A vs. B, or the time from onset to consultation ≤24 h vs. > 24 h, since data could not be obtained for a sufficient number of cases.

We could not perform meta-analyses on other symptoms like malaise or headache because most articles only reported the duration of overall symptoms and fever. An NRS by Nabeshima et al. in 2010 [25] reported the symptom severity of headache, malaise, chills, and myalgia/arthralgia on the consultation day (day 1) through day 6 in each group. They reported that the severity of headache was significantly better in the *maoto* group than in the NAIs group on days 1, 2, and 3. They also reported that the severity of myalgia/arthralgia was significantly better in the *maoto* group than in the NAIs group at night on day 1.

This NRS by Nabeshima et al. in 2010 [25] also reported that the dosing times of acetaminophen in their six-day study period were significantly reduced in the *maoto* group compared to in the oseltamivir group (0.6 ± 0.8 times in the *maoto* group vs. 2.4 ± 2.6 times in the oseltamivir group, *P* < 0.05 according to their analysis).

One RCT and one NRS reported on virus isolation by RADT after drug administration on days 3 and 5 [21, 29]. We did not find any significant differences in virus isolation between the groups.

#### Safety of the interventions

As shown in Table 2, data on the side effects and adverse reactions were available in eight of the identified studies [18–20, 22, 25, 28–30]. All reactions were mild, and no severe side effects or adverse reactions were reported as a result of either *maoto* or NAI administration. Only one study [22] reported morbidity. There were no cases of otitis media, bronchitis, pneumonia, febrile convulsion, or encephalitis. None of the studies reported mortality. Only one study [20] recorded hospitalisations; none of the patients were hospitalised.

**Table 2:** Side effects and adverse reactions

Study	Sample size			Side effects and adverse reactions in articles		
	*Maoto* + NAIs	NAIs	*Maoto*	*Maoto* + NAIs	NAIs	*Maoto*
Fukutomi 2005 [18]	10	12		None	None	
Kubo 2007 [20]	17	18		3 discontinued *maoto*	None	
Taketani 2008 [22]	40	145		None	None	
Tsuji 2011 [28]	28	30	23	2 cases of diarrhoea, 2 cases of vomiting, 1 case of abdominal pain, 1 case of mental excitement	1 case of diarrhoea, 1 cases of vomiting, 2 cases of abdominal pain, 2 cases of mental excitement, 1 case of dizziness, 1 case of periorbital oedema	2 cases of diarrhoea, 1 case of vomit
Toriumi 2012 [30]	89	124	58	11 discontinued treatment, 3 developed bacterial infection, 3 developed dehydration	4 discontinued treatment, 1 changed treatment, 3 cases of abnormal behaviour, 1 developed bacterial infection, 3 developed dehydration	5 discontinued treatment, 4 changed treatment, 3 developed bacterial infection
Kawamura 2007 [19]		43	86		10 cases of abnormal behaviour	9 cases of abnormal behaviour
Kawamura 2008 [21]		47	125		N/A	N/A
Kawamura 2009 [23]		33	97		N/A	N/A
Mori 2010 [24]		40	19		N/A	N/A
Nabeshima 2010 [25]		8	12		None	None
Suzuki 2011 [27]		46	40		N/A	N/A
Nabeshima 2012 [29]		22	11		1 case of mild transaminase elevation, 1 discontinued treatment	1 case of mild transaminase elevation

*NAI* neuraminidase inhibitor; None, no reported side effects or adverse reactions in the arm, *N/A* not available

Sample sizes differ from those in Additional file 2: Table S1 for studies including loss-to-follow-up-participants in the analysis of side effects

## Discussion

Although we could not reach a definitive conclusion because the included studies had a high risk of bias, the findings of this meta-analysis suggest that *maoto* may shorten the duration of fever when used alone or in combination with NAIs and that *maoto* may be a well-tolerated approach for alleviating flu symptoms. However, it should be noted that the quality of evidence for this conclusion was low. In the future, better-designed trials will be required to elucidate the efficacy of *maoto*.

We rated the quality of the evidence from the included studies as very low to low for a variety of reasons. First, only two RCTs were included, and the participants in the RCTs were assigned to each arm using open allocation. Second, we could not obtain enough information regarding the participants’ demographic backgrounds, including possible confounders such as vaccination history. Third, no blinded studies were identified via the database search conducted in the present review, although it would be difficult to perform a double-blinded study of *maoto* because it has a strong odour and taste [7]. Indeed, it would be difficult to design a perfect placebo for clinical trials, because the evaporating chemicals generate a characteristic odour, and this odour might affect the outcome [31]. Finally, none of the studies we employed in our analysis included a sample size calculation. Rather, the number of participants seemed to be based on the number of patients in the study season. Thus, we cannot exclude the possibility that β error occurred in the comparison between *maoto* and NAIs, where no significant difference was found according to the present analysis. To fully understand whether *maoto* is as effective as NAIs, an ‘equal efficacy’ or ‘non-inferiority’ study should be performed. It is worth performing such studies because *maoto* is much less expensive than are NAIs (approximately 20-fold in Japan) [15].

In the present study, we did not note any severe side effects or adverse reactions. Usually, *maoto* is consumed for several days, and we believe that the safety of this drug is high. However, it does contain ephedra, and clinicians should be cautious when prescribing it to patients who have thyroid, cardiovascular, or prostate dysfunction, or to elderly patients [32]. We acknowledge, however, that it may have been difficult for the researchers to collect comprehensive information regarding adverse events or complications, especially after the intervention period, because all of the studies we evaluated were performed in an outpatient setting. For example, the incidence of secondary bacterial pneumonia is common after the temporary resolution of influenza symptoms [33].

In future studies, *maoto* should ideally be used according to the traditional diagnosis of influenza, as mentioned in the introduction, and the inclusion or exclusion criteria should contain traditional concepts or diagnoses. However, what constitutes a traditional diagnosis may differ among researchers and clinicians, and it is difficult to utilise strictly defined criteria in clinical trials. In our previous study, none of the symptoms, such as chill and absence of sweating, were themselves obligate indicators for *maoto* in the alleviation of influenza symptoms, suggesting that *maoto* can be used to treat probable influenza infection regardless of whether traditional concepts or diagnoses are used [34].

Our search did not reveal any other reviews on *maoto*. However, the Cochrane collaboration did perform a systematic review of studies using traditional Chinese medicines, reporting that traditional Chinese medicines have clinical efficacy in alleviating influenza infection symptoms, similar to NAIs [7]. The systematic review by the Cochrane collaboration did not ultimately include one RCT by Kubo et al. [20] that was included in this review, because the intervention used in that RCT was *maoto,* a Japanese traditional medicine, not a traditional Chinese medicine.

## Conclusion

Even though a definitive conclusion could not be established owing to the small sample sizes and high risk of bias in the analysed studies, our findings imply that *maoto* may lower the fever duration when it is used alone or in combination with NAIs and is likely a well-tolerated treatment. However, the current evidence is too weak to draw a definitive conclusion. An RCT (if possible, double-blinded) must be conducted to determine the efficacy and safety of *maoto*. The present review and meta-analysis may be beneficial in estimating the effect size and calculating the sample size in future clinical trials.

## Additional files

Additional file 1: Search Strategies and Results. (DOCX 29 kb)

Additional file 2: **Table S1**. Basic characteristics of the included studies. (DOCX 27 kb)

Additional file 3: **Figure S1.** Funnel plot of fever duration after drug administration: *maoto* plus neuraminidase inhibitors vs. neuraminidase inhibitors alone. SE, size of effect; MD, mean difference. (PDF 35 kb)

Additional file 4: **Figure S2.** Funnel plot of overall symptom duration after drug administration: *maoto* vs. neuraminidase inhibitors. SE, size of effect; MD, mean difference. (PDF 38 kb)

Additional file 5: **Figure S3.** Funnel plot of fever duration after drug administration: *maoto* vs. neuraminidase inhibitors. SE, size of effect; MD, mean difference. (PDF 74 kb)

## Abbreviations

CENTRAL: Cochrane Central Register of Controlled Trials
CNKI: China National Knowledge Infrastructure
KAMJE: Korean Association of Medical Journal Editors
KMbase: Korean Medical database
NAI: Neuraminidase inhibitor
NRS: Non-randomised study
RADT: Rapid antigen detection test
RCT: Randomised controlled trial
WHO: World Health Organization
