# Supplementary material for: The use of maoto (Ma-Huang-Tang), a traditional Japanese Kampo medicine, to alleviate flu symptoms: a systematic review and meta-analysis
Source: BMC Complement Altern Med. 2019 Mar 18;19:68. doi: 10.1186/s12906-019-2474-z (PMC6421694; doi:10.1186/s12906-019-2474-z)
Supplement: Supplementary file 1 — Search Strategies and Results. (DOCX 29 kb) [file 12906_2019_2474_MOESM1_ESM.docx]

# English

The MEDLINE, CENTRAL and EMBASE search took the following format:

("influenza, human"[MeSH Terms] OR ("influenza"[All Fields] AND "human"[All Fields]) OR "human influenza"[All Fields] OR "flu"[All Fields]) AND ("maoto"[All Fields] OR "mao-to"[All Fields] OR ma-huang-tang[All Fields]).

Chinese:中国語

The CNKI（<http://www.cnki.net/>）and VIP(<http://lib.cqvip.com/index.aspx>) search took the following format:

("流行性感冒" OR “influenza” OR “flu”) AND

(“麻黄汤" OR “mahuang” OR “ma huang” OR "ma huang tang" OR "mahuang tang" OR “ma huang decoction” OR "mahuang decoction" OR “ephedra decoction”)

Search results

## CNKI

| \|  \| \| --- \| |
| --- | --- |
|  |

流行性感冒 AND 麻黄汤: 7

Two candidate articles were identified.

1) 扈晓宇、张扬、张德雄．经方本源剂量治疗流行性感冒风寒证、中国实验方剂学杂志．2011;17(13):230-234.

HU Xiao-yu, ZHANG Yang, ZHANG De-xiong, Original Dosage of Classical Prescription in Treating Influenza with Cold Syndrome

China Journal of Experimental Traditional Medical Formulae, 2011;17(13):230-234.

2) 马友全、白锋、刘占萍、韩小平．麻黄汤治疗太阳伤寒证的对照研究、中医临床研究．2011;3(7):34-35.

MA You-quan, Bai Feng, LIU Zhan-ping, HAN Xiao-ping, A control study on treating sun typhoid card with Mahuang decoction, Clinical Journal of Chinese Medicine, 2011;3(7):34-35,2011.

流行性感冒 AND mahuang tang :0

流行性感冒 AND ma huang tang: 0

流行性感冒 AND mahuang decoction :0

流行性感冒 AND ma huang decoction: 0

流行性感冒 AND ephedra decoction: 0

| \|  \| \| --- \| |
| --- | --- |
|  |

Influenza AND 麻黄汤: 4 (No relevant research of mahuangtang/maoto was added)

influenza AND mahuang tang: 0

influenza AND ma huang tang: 0

Influenza AND ma huang decoction: 0

influenza AND mahuang decoction: 11（No relevant research of mahuangtang/maoto was added）

influenza AND ephedra decoction: 1（No relevant research of mahuangtang/maoto was added）

|  |
| --- |

Flu AND 麻黄汤: 0

Flu AND mahuang tang: 0

Flu AND ma huang tang: 0

Flu AND mahuang decoction: 0

Flu AND ma huang decoction: 0

Flu AND ephedra decoction: 0

## VIP

流行性感冒 AND 麻黄汤:6

Three candidate articles were identified.

1) 李红娟、左俊岭．达菲联合中药治疗流行性感冒的临床观察、广州中医药大学学报．2014;31(5): 716-719.

LI Hongjuan, ZUO Junling. Clinical Observation of Oseltamivir Combined with Chinese Medicine for Treatment of Influenza. Journal of Guangzhou University of Traditional Chinese Medicine, 2014;31(5):716-719.

2) 扈晓宇、张扬、张德雄．经方本源剂量治疗流行性感冒风寒证、中国实验方剂学杂志．2011;17(13):230-234.

HU Xiao-yu, ZHANG Yang, ZHANG De-xiong,

Original Dosage of Classical Prescription in Treating Influenza with Cold Syndrome

China Journal of Experimental Traditional Medical Formulae, 2011;17(13):230-234.

3) 马友全、白锋、刘占萍、韩小平．麻黄汤治疗太阳伤寒证的对照研究、中医临床研究．2011;3(7):34-35.

MA You-quan, Bai Feng, LIU Zhan-ping, HAN Xiao-ping, A control study on treating sun typhoid card with Mahuang decoction, Clinical Journal of Chinese Medicine, 2011;3(7):34-35,2011.

流行性感冒 AND mahuang tang: 0

流行性感冒 AND ma huang tang: 0

流行性感冒 AND mahuang decoction: 1 (Ma 2001)

流行性感冒 AND ma huang tang: 0

流行性感冒 AND ephedra decoction: 0

Influenza AND 麻黄汤:5 (No relevant research of mahuangtang/maoto was added)

Influenza AND mahuang tang: 3 (No relevant research of mahuangtang/maoto was added)

Influenza AND ma huang tang: 0

Influenza AND mahuang decoction: 8 (No relevant research of mahuangtang/maoto was added)

Influenza AND ma huang decoction: 0

Influenza AND ephedra decoction: 3 (No relevant research of mahuangtang/maoto was added)

Flu AND 麻黄汤: 0

Flu AND mahuang tang: 0

Flu AND ma huang tang: 0

Flu AND mahuang decoction: 0

Flu AND ma huang decoction: 0

Flu AND ephedra decoction: 0

# Korean:韓国語

The KMbase (<http://en.medric.or.kr/> or <http://medric.or.kr/>)

and KoreaMed (<https://koreamed.org/SearchBasic.php>) search took the following format:

(“influenza” OR “인플루엔자”(インフルエンザ) OR “독감”(毒感)) AND

(“mahwang-tang” “ma huang tang”OR “마황탕 (麻黄湯) ”)

Search results

- KMbase (In English)

influenza AND mahwang-tang: 0

influenza AND ma huang tang: 0

influenza AND 마황탕: 0

인플루엔자 AND mahwang-tang: 0

인플루엔자 AND ma huang tang: 0

인플루엔자 AND 마황탕: 0

독감 AND mahwang-tang: 0

독감 AND ma huang tang: 0

독감 AND마황탕: 0

- KAMJE

influenza AND mahwang-tang: 0

influenza AND ma huang tang: 0

influenza AND 마황탕: 0

인플루엔자 AND mahwang-tang: 0

인플루엔자 AND ma huang tang: 0

인플루엔자 AND 마황탕: 0

독감 AND mahwang-tang: 0

독감 AND ma huang tang: 0

독감 AND마황탕: 0
