# Supplementary material for: The use of maoto (Ma-Huang-Tang), a traditional Japanese Kampo medicine, to alleviate flu symptoms: a systematic review and meta-analysis
Source: BMC Complement Altern Med. 2019 Mar 18;19:68. doi: 10.1186/s12906-019-2474-z (PMC6421694; doi:10.1186/s12906-019-2474-z)
Supplement: Supplementary file 2 — Table S1. Basic characteristics of the included studies. (DOCX 27 kb) [file 12906_2019_2474_MOESM2_ESM.docx]

**Additional file 2: Table S1.** Basic characteristics of the included studies

| Study | Studied in | Manuscript  language | Sample size | | | Female sex | | | Age (years) | | | Background table | Label |
| --- | --- | --- | --- | --- | --- | --- | --- | --- | --- | --- | --- | --- | --- |
|  |  |  | *Maoto*  + NAIs | NAIs | *Maoto* | *Maoto*  + NAIs | NAIs | *Maoto* | *Maoto*  + NAIs | NAIs | *Maoto* |  |  |
| Fukutomi 2005 [18] | Japan | Japanese | 10 | 12 |  | 4 | 8 |  | 33.6 ± 10.4 | 31.9 ± 13.0 |  | Yes | PCS |
| Kubo 2007 [20] | Japan | English | 14 | 18 |  | 8 | 8 |  | 5.3 ± 2.0 | 5.4 ± 2.0 |  | Yes | RCT |
| Taketani 2008 [22] | Japan | Japanese | 40 | 145 |  | 17 | 70 |  | 0.6–13.3 | 0.5–14.8 |  | Yes | PCS |
| Tsuji 2011 [28] | Japan | Japanese | 28 | 30 | 23 | N/A | N/A | N/A | N/A | N/A | N/A | No | RCS |
| Toriumi 2012 [30] | Japan | English | 66 | 108 | 46 | 37 | 54 | 21 | 7.5 ± 3.6 | 8.3 ± 3.9 | 7.8 ± 4.7 | Yes | PCS |
| Kawamura 2007 [19] | Japan | Japanese |  | 49 | 86 |  | 24 | 36 |  | 8.5 ± 9.0 | 7.5 ± 6.6 | Yes | PCS |
| Kawamura 2008 [21] | Japan | Japanese |  | 47 | 125 |  | 25 | 63 |  | 6.4 ± 7.2 | 7.8 ± 7.4 | Yes | PCS |
| Kawamura 2009 [23] | Japan | Japanese |  | 33 | 97 |  | 18 | 46 |  | 12.1 ± 16.0 | 8.2 ± 8.9 | Yes | PCS |
| Mori 2010 [24] | Japan | Japanese |  | 40 | 19 |  | 22 | 7 |  | 15.9 ± 12.6 | 11 ± 11 | Yes | RCS |
| Nabeshima 2010 [25] | Japan | English |  | 8 | 12 |  | 3 | 4 |  | 35.8 ± 14.0 | 23.4 ± 10.5 | Yes | PCS |
| Suzuki 2011 [27] | Japan | Japanese |  | 46 | 40 |  | 18 | 16 |  | 5.1 ± 2.2 | 5.4 ± 2.5 | Yes | Quasi-RCT |
| Nabeshima 2012 [29] | Japan | English |  | 18 | 10 |  | 10 | 4 |  | 28.4 ± 11.2 | 29.2 ± 12.7 | Yes | RCT |

**Table 1.** Basic characteristics of the included studies (Continued)

| Study | Diagnosis | Time from onset to consultation | Daily dosage of *maoto* | Pharmaceutical company of *maoto* | Name of NAI and daily dosage | Duration of intervention (days) | |
| --- | --- | --- | --- | --- | --- | --- | --- |
|  |  |  |  |  |  | *Maoto* | NAI |
| Fukutomi 2005 | Flu by RADT | ≤24 h | 7.5 g/day | Extract by Tsumura | Oseltamivir 150 mg/day | N/A | N/A |
| Kubo 2007 | Flu A by RADT + PCR/culture | ≤48 h | 0.06 g/kg/day | Extract by Tsumura | Oseltamivir 4 mg/kg/day | N/A | N/A |
| Taketani 2008 | Flu by RADT | ≤48 h | 0.15–0.20 g/kg/day | Extract by Tsumura | Oseltamivir 4 mg/kg/day | N/A | N/A |
| Tsuji 2011 | Flu by RADT | N/A | 0.2 g/kg/day | Extract by Tsumura | Oseltamivir 4 mg/kg/day Zanamivir 10 mg/day | 5 | 5 |
| Toriumi 2012 | Flu by RADT | ≤48 h | 0.06 g/kg/day | Extract by Tsumura | Oseltamivir 4 mg/kg/day Zanamivir 10 mg/day | N/A | N/A |
| Kawamura 2007 | Flu by RADT | No restriction | 0.08–0.19 g/kg/day, max 7.5 g/day | Extract by Tsumura | Oseltamivir 4 mg/kg/day,  max 150 mg/day | 2–8 | 2–5 |
| Kawamura 2008 | Flu A by RADT | No restriction | 0.16 g/kg/day,  max 7.5 g/day | Extract by Tsumura or Tetikoku | Oseltamivir 3.9 mg/kg/day | 1–6 | ~5 |
| Kawamura 2009 | Flu A by RADT + PCR | ≤48 h | 0.2 g/kg/day,  max 7.5 g/day | Extract by Teikoku | Oseltamivir 4 mg/kg/day | 1–5 | ~5 |
| Mori 2010 | Flu A by RADT | N/A | 2.5 g/2 h, then 2.5 g/3 h until sweating began | Extract by Tsumura | Oseltamivir N/A Zanamivir N/A | N/A | N/A |
| Nabeshima 2010 | Flu A by RADT | ≤48 h | 7.5 g/day | Extract by Tsumura | Oseltamivir 150 mg/day | 5 | 5 |
| Suzuki 2011 | Flu A by RADT | N/A | 0.15 g/kg/day | Extract by Tsumura | Oseltamivir 4 mg/kg/day | 5 | 5 |
| Nabeshima 2012 | Flu by RADT | ≤48 h | 7.5 g/day | Extract by Tsumura | Oseltamivir 150 mg/day | 5 | 5 |

**Table 1.** Basic characteristics of the included studies (Continued)

| Study | Duration of symptoms | | | | | |
| --- | --- | --- | --- | --- | --- | --- |
|  | Overall symptoms  (mean ± SD; unit is hours unless otherwise stated) | Fever  (mean ± SD; unit is hours unless otherwise stated, definition of fever follows) | Headache | Malaise | Myalgia | Chills |
| Fukutomi 2005 | N/A | *Maoto* + NAI: 1.9 ± 0.6 days  NAI: 1.7 ± 0.8 days  ≥38°C | *Maoto* + NAI: 1.3 ± 0.5 days  NAI: 2.4 ± 1.0 days | *Maoto* + NAI: 1.3 ± 0.5 days  NAI: 2.3 ± 1.2 days | N/A | N/A |
| Kubo 2007 | N/A | *Maoto* + NAI: median 18 [95%CI 15.2–27.7]  NAI: Median 24 [95%CI 23.5–43.0]  ≥37.2°C | N/A | N/A | N/A | N/A |
| Taketani 2008 | N/A | *Maoto* + NAI: 16.64 ± 11.28  NAI: 21.53 ± 14.6  ≥37.5°C | N/A | N/A | N/A | N/A |
| Tsuji 2011 | *Maoto* + NAI: 4.5 ± 1.3 days  NAI: 4.8 ± 1.4 days  *Maoto*: 5.1 ± 1.8 days | *Maoto* + NAI: 57.1 ± 25.4  NAI: 66.5 ± 31.0  *Maoto*: 74.4 ± 41.8  N/A | N/A | N/A | N/A | N/A |
| Toriumi 2012 | N/A | *Maoto* + NAI: 34.01 ± 25.18  NAI: 43.45 ± 31.15  *Maoto*: 38.9 ± 30.15  ≥37.5°C | N/A | N/A | N/A | N/A |
| Kawamura 2007 | NAI: 69.21 ± 36.00  *Maoto*: 54.55 ± 34.64 | NAI: 33.20 ± 22.34  *Maoto*: 34.20 ± 27.33  ≥37.5°C | N/A | N/A | N/A | N/A |
| Kawamura 2008 | N/A | NAI: 30.36 ± 20.96  *Maoto*: 49.72 ± 37.54  ≥37.5°C | N/A | N/A | N/A | N/A |
| Kawamura 2009 | NAI: 47.15 ± 31.67  *Maoto*: 47.23 ± 30.37 | NAI: 40.82 ± 28.92  *Maoto*: 42.40 ± 32.3  ≥37.5°C | N/A | N/A | N/A | N/A |
| Mori 2010 | N/A | NAI: 22.0 ± 9.6  *Maoto*: 17.4 ± 7.7  ≥37°C | N/A | N/A | N/A | N/A |
| Nabeshima 2010 | NAI: 84.4 ± 44.1  *Maoto*: 80.8 ± 36.8 | NAI: 20.0 ± 10.3  *Maoto*: 21.4 ± 19.8  ≥37.5°C | Data only in figure format | Data only in figure format | Data only in figure format | Data only in figure format |
| Suzuki 2011 | N/A | NAI: 67.0 ± 24.4  *Maoto*: 84.8 ± 36.8  ≥37.5°C | N/A | N/A | N/A | N/A |
| Nabeshima 2012 | NAIs: Oseltamivir median 87  Zanamivir median 94  *Maoto*: Median 83 | NAIs: Oseltamivir median 46  Zanamivir median 27  *Maoto*: Median 29  ≥37.5°C | N/A | N/A | N/A | N/A |

Table 1 Basic characteristics of the included studies (Continued)

| Study | Dosing times of acetaminophen  (times) | Virus isolation on day 3  (number of patients) | Virus isolation on day 5  (number of patients) |
| --- | --- | --- | --- |
| Fukutomi 2005 | N/A | N/A | N/A |
| Kubo 2007 | N/A | N/A | N/A |
| Taketani 2008 | N/A | N/A | N/A |
| Tsuji 2011 | N/A | N/A | N/A |
| Toriumi 2012 | N/A | N/A | N/A |
| Kawamura 2007 | N/A | N/A | N/A |
| Kawamura 2008 | N/A | NAI: 17/47  *Maoto*: 54/125 | NAI: 5/47  *Maoto*: 11/125 |
| Kawamura 2009 | N/A | N/A | N/A |
| Mori 2010 | N/A | N/A | N/A |
| Nabeshima 2010 | NAI: 2.4 ± 2.6 (in six days)  *Maoto*: 0.6 ± 0.8 (in six days) | N/A | N/A |
| Suzuki 2011 | N/A | N/A | N/A |
| Nabeshima 2012 | N/A | NAI: 5/11  *Maoto*: 4/7 | NAI: 2/11  *Maoto*: 1/7 |

NAI, neuraminidase inhibitor; PCS, prospective cohort study; RCT, randomised controlled trial; N/A, not available; RCS, retrospective cohort study; RADT, rapid antigen detection test; PCR, polymerase chain reaction; SD, standard deviation; CI, confidence interval
