# Supplementary figures and images for: The use of maoto (Ma-Huang-Tang), a traditional Japanese Kampo medicine, to alleviate flu symptoms: a systematic review and meta-analysis
Source: BMC Complement Altern Med. 2019 Mar 18;19:68. doi: 10.1186/s12906-019-2474-z (PMC6421694; doi:10.1186/s12906-019-2474-z)

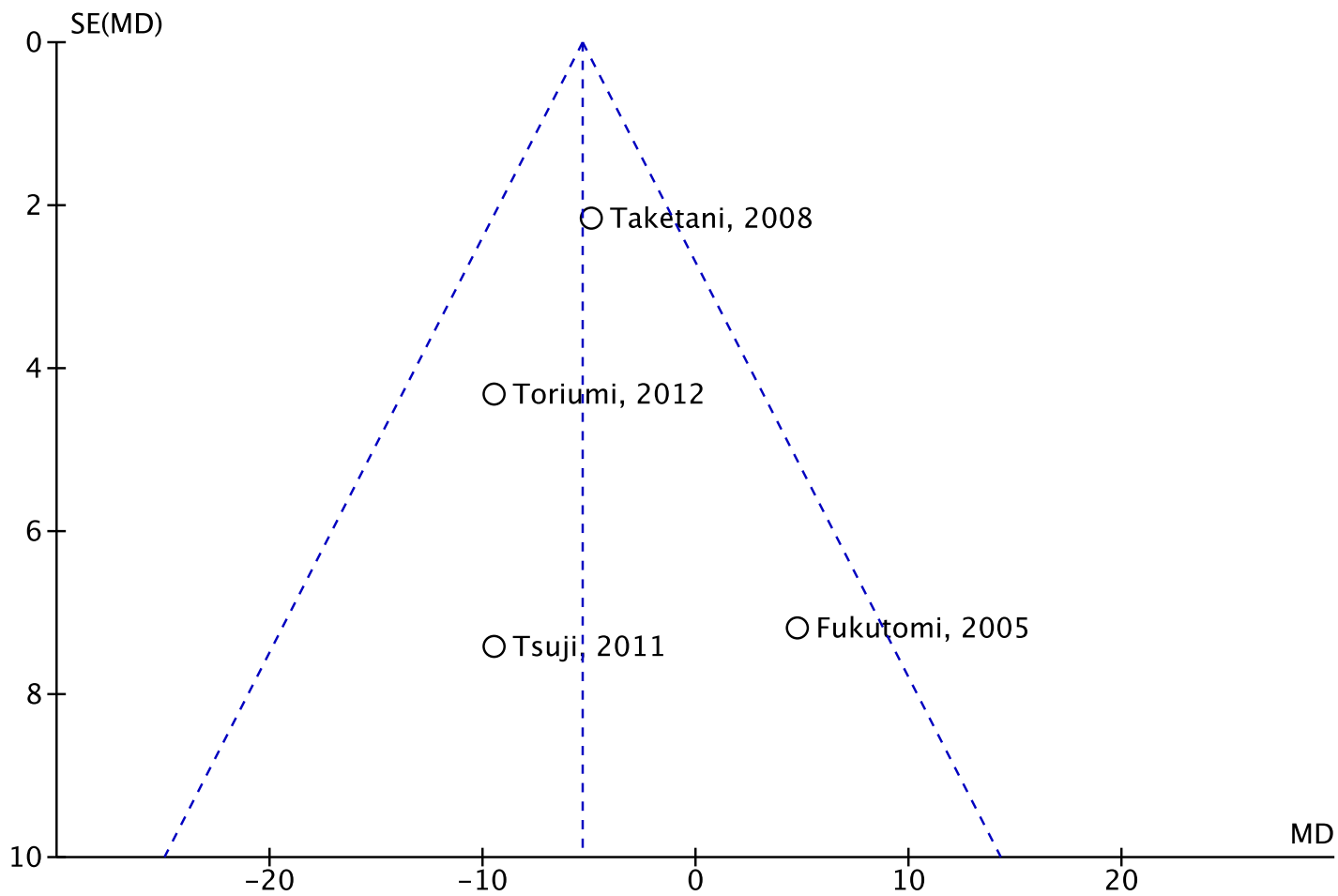

Supplement: Supplementary file 3 — Figure S1. Funnel plot of fever duration after drug administration: maoto plus neuraminidase inhibitors vs. neuraminidase inhibitors alone. SE, size of effect; MD, mean difference. (PDF 35 kb) [file 12906_2019_2474_MOESM3_ESM.pdf]

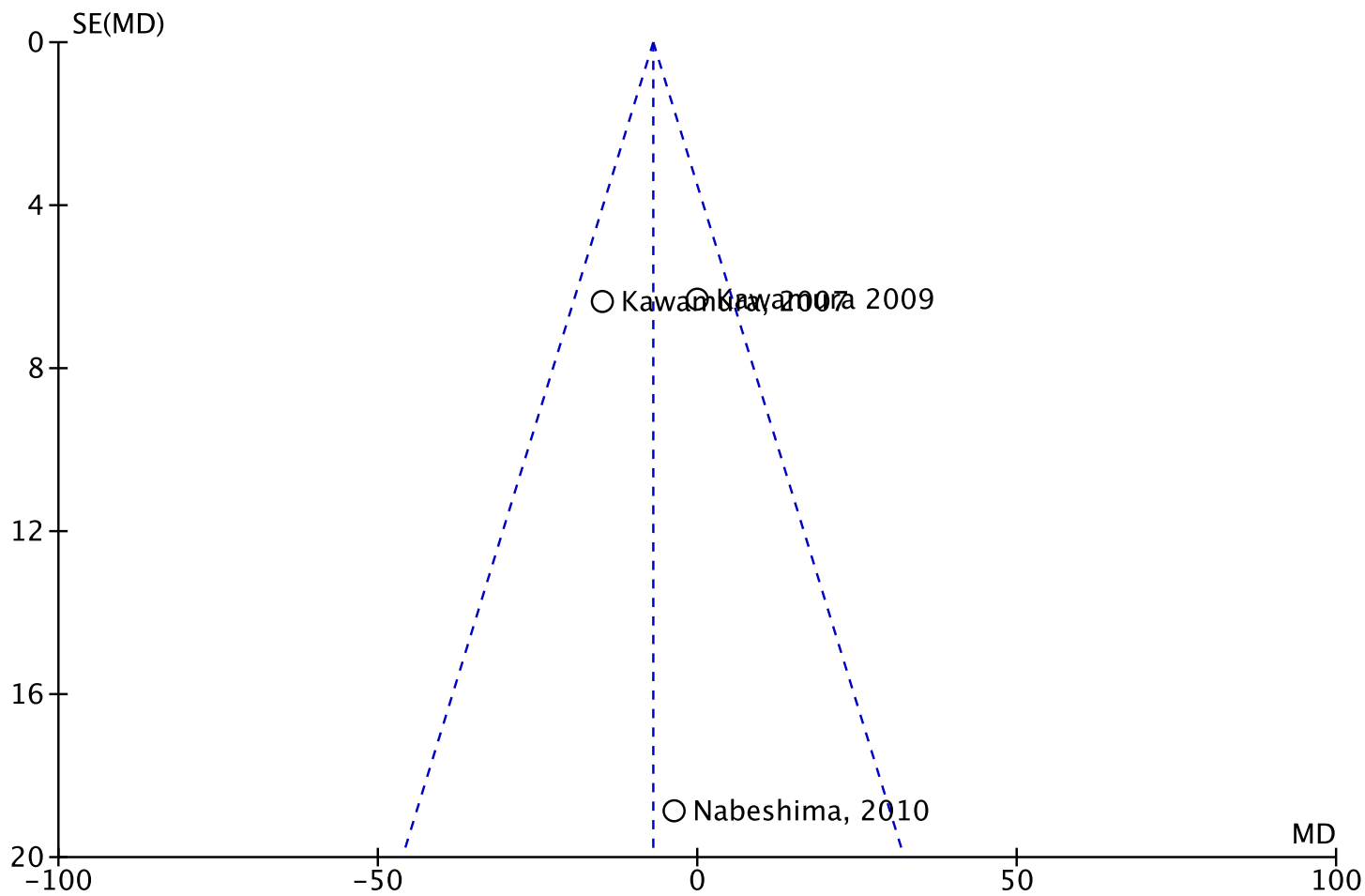

Supplement: Supplementary file 4 — Figure S2. Funnel plot of overall symptom duration after drug administration: maoto vs. neuraminidase inhibitors. SE, size of effect; MD, mean difference. (PDF 38 kb) [file 12906_2019_2474_MOESM4_ESM.pdf]

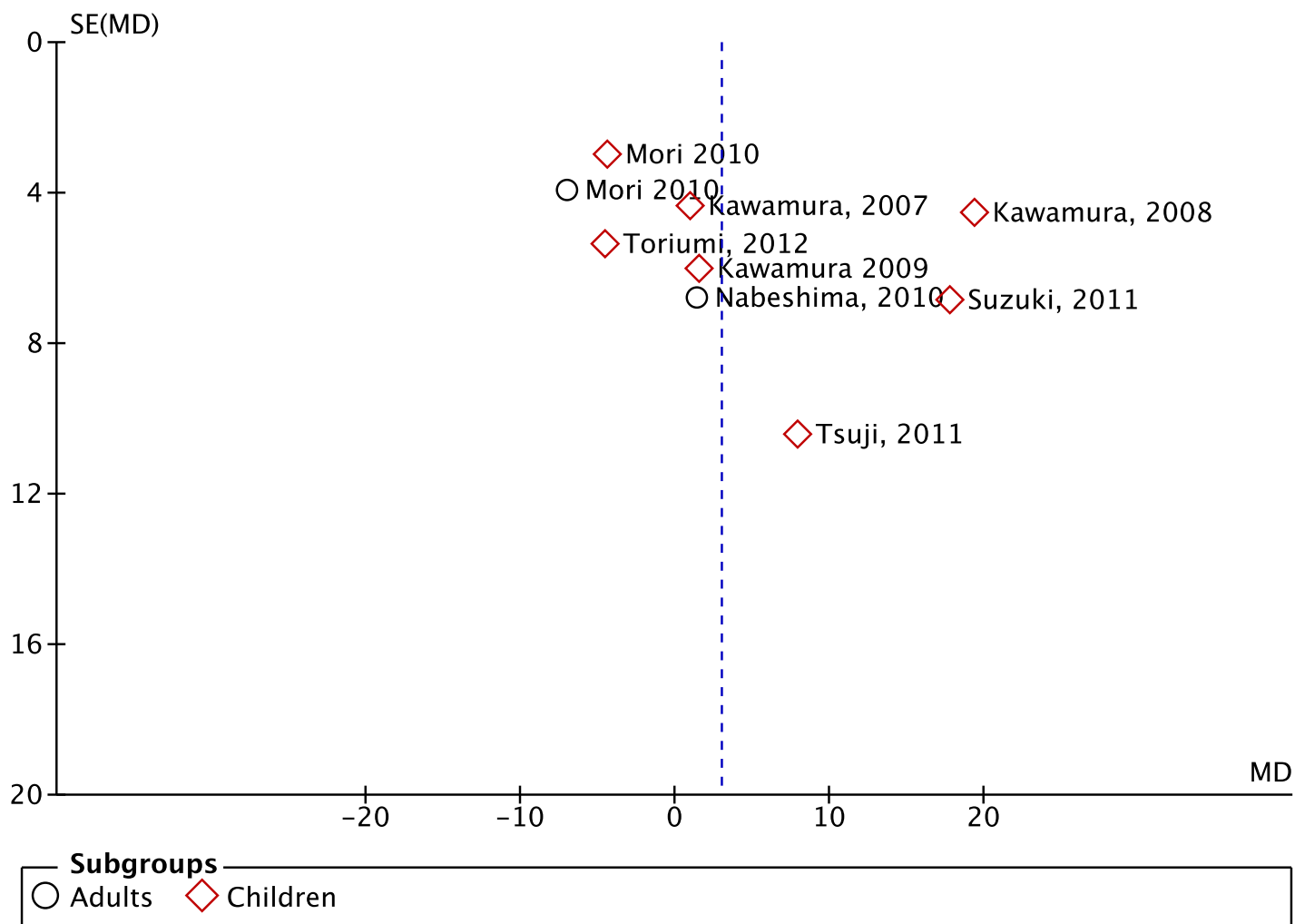

Supplement: Supplementary file 5 — Figure S3. Funnel plot of fever duration after drug administration: maoto vs. neuraminidase inhibitors. SE, size of effect; MD, mean difference. (PDF 74 kb) [file 12906_2019_2474_MOESM5_ESM.pdf]
